# Radiomic analysis of high-resolution computed tomography predicts interstitial lung disease progression and mortality in systemic sclerosis

**DOI:** 10.1093/rheumatology/keag463

**Published:** 2026-08-27

**Authors:** Maria Iacovantuono, Nicholas Landini, Lisa Jungblut, Gesa M Sauer, Florian Käs, Rucsandra Dobrota, Sinziana Muraru, Muriel Elhai, Carina Mihai, Mike O Becker, Maria Sole Chimenti, Thomas Frauenfelder, Anna-Maria Hoffmann-Vold, Oliver Distler, Cosimo Bruni

**Affiliations:** Department of Rheumatology, University Hospital Zurich, University of Zurich, Zurich, Switzerland; Department of Systems Medicine, Rheumatology, Allergology and Clinical Immunology, University of Rome Tor Vergata, Rome, Italy; Department of Radiological, Oncological and Pathological Sciences, “Sapienza” University of Rome, Policlinico Umberto I, Rome, Italy; Institute for Diagnostic and Interventional Radiology, University Hospital Zurich, University Zurich, Zurich, Switzerland; Department of Rheumatology, University Hospital Zurich, University of Zurich, Zurich, Switzerland; Department of Rheumatology, University Hospital Zurich, University of Zurich, Zurich, Switzerland; Department of Rheumatology, University Hospital Zurich, University of Zurich, Zurich, Switzerland; Department of Rheumatology, University Hospital Zurich, University of Zurich, Zurich, Switzerland; Department of Rheumatology, University Hospital Zurich, University of Zurich, Zurich, Switzerland; Department of Rheumatology, University Hospital Zurich, University of Zurich, Zurich, Switzerland; Department of Rheumatology, University Hospital Zurich, University of Zurich, Zurich, Switzerland; Department of Systems Medicine, Rheumatology, Allergology and Clinical Immunology, University of Rome Tor Vergata, Rome, Italy; Institute for Diagnostic and Interventional Radiology, University Hospital Zurich, University Zurich, Zurich, Switzerland; Department of Rheumatology, University Hospital Zurich, University of Zurich, Zurich, Switzerland; Department of Rheumatology, Oslo University Hospital, Oslo, Norway; Department of Rheumatology, University Hospital Zurich, University of Zurich, Zurich, Switzerland; Department of Rheumatology, University Hospital Zurich, University of Zurich, Zurich, Switzerland

**Keywords:** systemic sclerosis, interstitial lung disease, radiomics, ILD progression, mortality

## Abstract

**Objective:**

The automated assessment of chest high-resolution CT (HRCT) scans allows the objective quantification of interstitial lung disease (ILD)–related features. Here, we explored the ability of HRCT-derived radiomic features to predict all-cause mortality and ILD progression in SSc-ILD patients.

**Methods:**

We analysed baseline and follow-up HRCT scans of SSc-ILD patients using lung texture analysis (LTA™, Imbio). Lung parenchymal alterations (honeycombing, ground-glass, reticulation, hyperlucency) and pulmonary vessel volume (PVV) were quantified as percentages of the respective lung volume—for whole lungs and for upper, middle and lower zones. Univariable and multivariable Cox regression and generalized estimating equation (GEE) models were applied to identify radiomics predictors of all-cause mortality and ILD progression, respectively.

**Results:**

Of 313 SSc-ILD patients, 267 (85%) were eligible for radiomic analysis and were included in the mortality analysis; 160 (60%) were included in the progression analysis. Over 42 [interquartile range (IQ)R 27–110] months of follow-up, 65 (24%) patients died. They presented with a greater extent of PVV% and parenchymal alterations across all lung zones, compared with patients who survived. PVV% independently predicted mortality [adjusted hazard ratio (HR) 1.21, 95% CI 1.094–1.354], particularly in the upper zones (adjusted HR 1.28, 95% CI 1.11–1.46). Among 261 yearly follow-up visits from 160 SSc-ILD patients, 50.6% showed at least one episode of ILD progression, for a total of 99 (37.7%) episodes. Baseline radiomic features were overall comparable between progressors and non-progressors. The extent of whole-lung honeycombing was identified as the only independent radiomic predictor of ILD progression [adjusted odds ratio 1.53, 95% CI 1.17–2.01].

**Conclusion:**

The PVV% and the extent of honeycombing independently predict mortality and ILD progression, respectively. These radiomic features might support risk stratification of SSc-ILD patients, on top of functional and clinical characteristics.

## Graphical abstract

**Figure:**
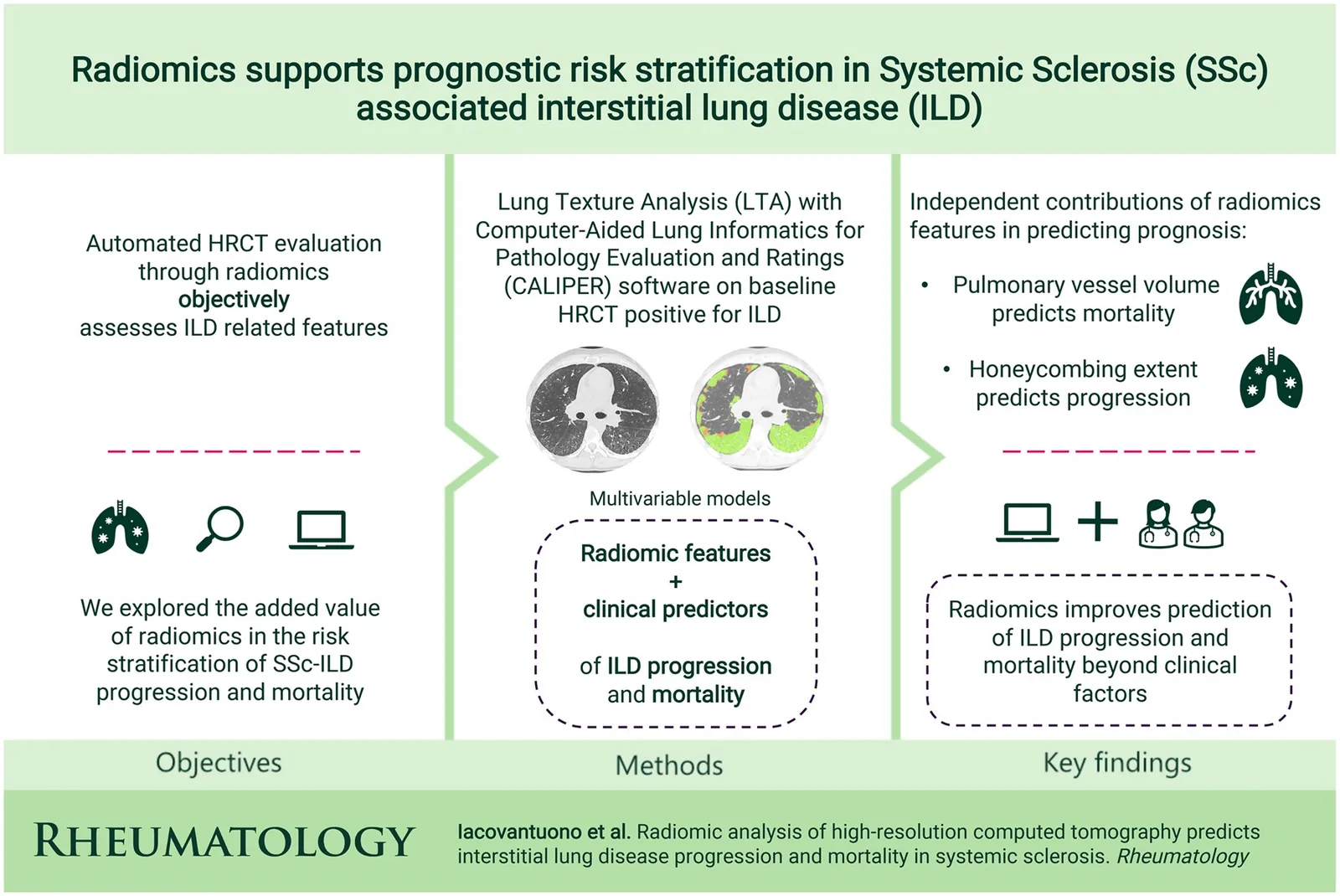

*Rheumatology* key messages

- Quantitative assessment of lung pathology and vessel volume predicts prognosis in SSc-ILD.
- Radiomics increased pulmonary vessel volume predicts mortality.
- Radiomics honeycombing alterations extent predicts ILD progression.

## Introduction

Interstitial lung disease (ILD) is highly prevalent in patients with SSc, occurring in at least 50% of cases [1] and strongly impacting all-cause mortality [2], especially when progressive [3]. SSc-ILD progression is extremely heterogeneous over time [3], complicating the identification and validation of risk factors associated with poor prognosis [2, 4, 5]. As a result, risk stratification remains suboptimal [6, 7]. High-resolution CT (HRCT) plays a major role in ILD diagnosis and monitoring and is also recommended as a screening tool [8]. Although the visual quantification of lung involvement has been linked with both progression and mortality in SSc-ILD [9], its applicability in research and in clinical practice is impaired by limited reproducibility, the need for expert assessors and the lack of standardized definitions. In this context, radiomics has emerged as a promising approach for the objective and quantitative assessment of imaging data beyond visual interpretation. Radiomics enables the extraction of high-dimensional features that capture subtle structural and textural characteristics related to underlying pathophysiological processes, enhancing a reproducible quantification of pulmonary abnormalities from conventional imaging [10]. By converting medical images into high-dimensional data, radiomics offers substantial potential for advancement in the field of ILD [11]. However, the application of radiomics in SSc-ILD is still limited. While some smaller studies have identified ILD extent as a risk factor for mortality [9], others have explored quantitative imaging measures to assess therapeutic response. In addition, imaging measures have been correlated with functional decline, although this has not been consistently evaluated according to standardized criteria [12]. As a result, evidence on radiomics-based prediction of ILD progression remains limited and methodologically heterogeneous.

The interplay between parenchymal alterations and pulmonary vasculature detected through radiomic analysis of HRCTs has emerged as a potential compensatory mechanism in SSc-ILD [13]. The increased pulmonary vessel volume (PVV) within less fibrotic areas has been linked to the presence and the severity of SSc-ILD, paralleled by a phenomenon of vascular redistribution from the more fibrotic to the less affected areas [14]. In addition, the extent of PVV (PVV%) has been shown to predict survival and functional lung decline in idiopathic pulmonary fibrosis (IPF) and RA-associated ILD [15, 16]. This suggests that vascular-related imaging features may provide prognostic information beyond parenchymal extent alone.

Given the major prognostic impact of SSc-ILD and the availability of effective therapies, profiling patients at risk of poor prognosis is essential for optimizing therapeutic management, improving survival, and achieving personalized strategies [17]. A clearer understanding of how established risk factors integrate with imaging-derived biomarkers is needed to better stratify patients. Following the hypothesis that radiomics can identify key features of ILD not detectable on visual assessment, the current study aimed to investigate the potential contribution of radiomic markers in the prognosis and risk stratification of SSc-ILD.

## Methods

### Patients’ characteristics and inclusion criteria

We designed a monocentric, retrospective and longitudinal cohort study, including SSc patients fulfilling the 2013 EULAR/ACR classification criteria [18], attending the Department of Rheumatology, University Hospital of Zurich, from 2003 to 2023 and undergoing at least one HRCT scan. To be eligible for the study, patients required presence of ILD on HRCT confirmed by experienced thoracic radiologists, blinded to clinical outcomes (N.L., L.J.), and technical suitability for the radiomic analysis (see radiomic analysis section below).

In case of multiple HRCT scans suitable for the same patient, the date of the first eligible HRCT was defined as the baseline scan. For each assessable time point, demographic, clinical, and laboratory findings were collected in line with the European Scleroderma Trials and Research group (EUSTAR) definitions [19].

### Radiomic analysis

All retrieved HRCT examinations were performed at the University Hospital of Zurich. HRCT with motion artefacts were excluded by the evaluating thoracic radiologists. All images were acquired at the end inspiratory phase, without contrast enhancement. Each HRCT scan was assessed for technical suitability for the radiomic analysis. Reconstruction parameters required mediastinal window, with slice thickness and inter-slice spacing ≤2 mm, representing the upper limit for soft- convolution kernel reconstructions, excluding non-recommended kernels for the different scanners by the producing company software user manual [20]. This thorough selection process allowed standardized, harmonized, and homogenized HRCT selection across different scanners, years, and imaging protocols.

**Figure 1:**
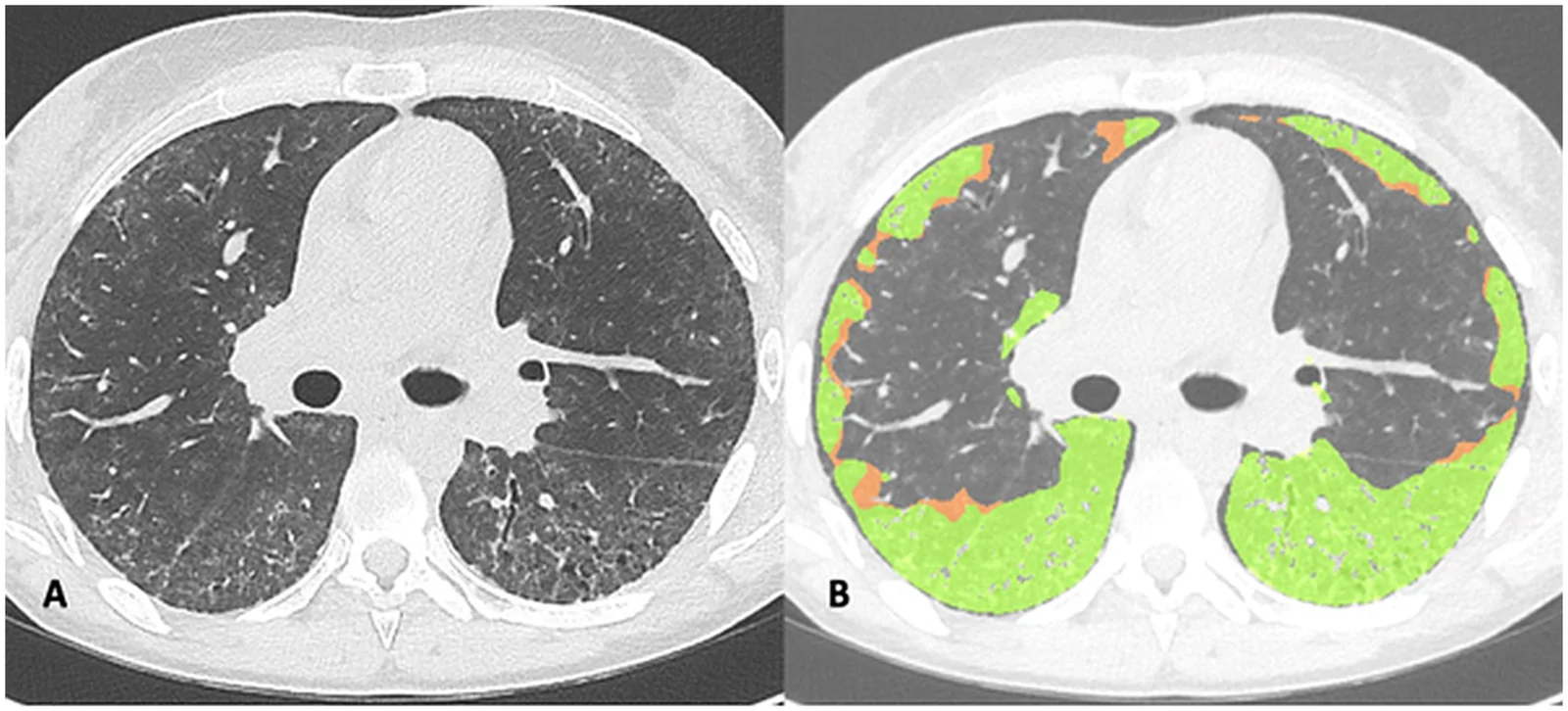
SSc-ILD on HRCT: visual assessment (A) and radiomic segmentation masks (B). HRCT: high-resolution CT; ILD: interstitial lung disease

Each eligible scan was post-processed for the quantitative examination, using the Imbio *lung texture analysis* (LTA) system, based on Computer-Aided Lung Informatics for Pathology Evaluation and Ratings—CALIPER- technology (Biomedical Imaging Resource, Mayo Clinic, Rochester, MN, USA). LTA automatically extracts the features from the whole lung after removal of the central airways, and segments it in the right and left upper, middle and lower zones, based on thirds. Six distinct radiomic features were quantified: normal lung, ground-glass opacities (GGOs), reticulation, hyperlucency, honeycombing, and PVV. An example of CT imaging and LTA corresponding to LTA assessment is presented in Fig. 1. From each lung third, the right and left data were merged to obtain the mean values of the upper, middle and lower zones. The volume of each feature was then expressed as a percentage of the respective lung volume. Given the known association of visual ILD extent and prognostic outcomes, we also analysed the association of traditional markers, i.e. ILD extent (the sum of GGOs, reticulation, and honeycombing) and fibrotic extent (the sum of reticulation and honeycombing), with progression and mortality.

### Definition of prognostic measures

For the survival analysis, we considered date of death or last follow-up visit with alive status.

For the progression analysis, we included patients with at least two consecutive visits 12 ± 3 months apart, at least one HRCT scan suitable for the radiomic analysis at the first visit of the interval, and pulmonary function tests (PFTs) available at both time points. No HRCT radiomic evaluation was required at the end of the time interval for progression assessment. For each patient, multiple intervals could be included, only with the data and CT information at the start of the 1-year period being included in the model.

ILD progression between two consecutive visits was defined according to the physiological domain of the Progressive Pulmonary Fibrosis (PPF)-ILD criteria, as absolute forced vital capacity (FVC) decline of ≥5% or diffusion of the lung for carbon oxide (DLCO) absolute decline of ≥10%, after excluding other causes, as pulmonary hypertension (PH) deterioration [20]; in line with the same criteria, appropriate differential diagnosis was performed when assessing for progression (i.e. from intercurrent infection) through medical record evaluation.

Additional definitions of ILD progression were also tested as independent outcomes, including:

- FVC absolute decline of ≥5% [3];
- FVC relative decline of ≥10% or FVC relative decline of 5%–9% and DLCO relative decline of ≥15% [21, 22];
- FVC relative decline of ≥10% [23].

### Covariates

Clinical risk factors derived from the literature and expert opinion were used to adjust the independent contribution of the radiomic features in the prediction models. For the survival analysis, the Scleroderma mOrtality p Eustar (SCOpE) was included as a multifactorial, all-cause mortality risk factor [2]. The SCOpE score includes 13 clinical variables, such as age, male sex, cutaneous disease subset, elevated CRP levels, NYHA functional class ≥ II, presence of ILD, DLCO and FVC % predicted values, proteinuria, scleroderma renal crisis, left ventricular ejection fraction, digital ulcers, and joint involvement. For the progression analysis, age, sex, disease duration from the first non-RP sign or symptoms, dyspnoea NYHA class, baseline DLCO%, baseline FVC%, and diffuse skin involvement were selected as adjustment variables [24].

### Statistical analysis

Mean ± standard deviation or median and interquartile ranges (IQRs) were reported to describe continuous variables, according to their distribution. Absolute and relative frequencies were reported for categorical and dichotomous variables. The unpaired Student’s *t* test, χ^2^ or the Fisher exact test were used to compare clinical, functional and radiomics characteristics between groups.

To independently assess the association of radiomics-derived parameters with mortality, we first created a multivariable Cox proportional hazards models with backward selection including only radiomic features of the baseline HRCT. Significant radiomic predictors were then combined into a second multivariable model adjusted for the SCOpE score to test their independent contribution. When a significant radiomic predictor was detected for the whole-lung LTA analysis, a sensitivity analysis was performed including the same feature in the upper, middle and lower zones, respectively, to assess the consistency of these findings across the whole lung.

For the progression analysis, similarly, we first applied a multivariable regression model with backward selection to identify significant radiomic predictors. These were then combined into a second multivariable model with the abovementioned confounders, to determine their independent contribution. To account for multiple observations of the same patient, we adopted a Generalized Estimating Equation Model (GEE). Again, sensitivity analyses for the separate lung thirds were performed in cases of significant results of the whole lung analysis.

A complete case analysis was conducted using all available data. The multivariable models adhered to the 1:10 rule of thumb [25], requiring ∼10 events per independent variable tested. Acceptable collinearity among predictors was assessed using the Variance Inflation Factor (VIF), with a threshold of five. Data analysis was performed with IBM SPSS v29.0.0.0, with statistical significance defined as a two-sided *P*-value of <0.05.

## Results

Of 314 patients with ILD at enrolment in the registry, 47 (15%) patients were excluded because their HRCT scans were not suitable for radiomic analysis.

Of the 267 (85%) patients with SSc-ILD eligible for the study, 204 (76.4%) were female, the mean age was 59 (s.d. 14.2) years and the median disease duration was 5.9 (1.9–15) years. Anti-topo I antibodies (ATA) were positive in 119 (44.7%) patients, and 92 (34.5%) had diffuse cutaneous involvement (dcSSc).

### All-cause mortality

All the 267 SSc-ILD included patients were eligible for the mortality analysis. Over a median follow-up of 42 (IQR 27–110) months, a total of 65 (24%) deaths were recorded. SSc-ILD patients who died were older at baseline, had more increased inflammatory markers, were more likely to be smokers, and presented more severely impaired PFTs compared with those who survived (Table 1). The use of immunosuppressive medications (MMF, CYC, rituximab or tocilizumab) or nintedanib [26] was also more frequent among patients who died. Comparing the radiomic features between the two groups, patients who died presented with a higher extent of all radiomic parameters at baseline, including PVV, both in whole lung and across all lung fields (Table 2, left panel).

**Table 1:** Clinical characteristics and functional features of SSc patients with death or alive status at the end of follow-up. Increased inflammatory markers are defined as CRP or ESR above the upper level of normality according to local laboratory. Immunosuppressants include CYC, MMF, rituximab, tocilizumab.

	Total *n* = 267	**Death *n* (%)** **=** **65 (24.3)**	**Alive *n* (%)** **=** **202 (75.7)**	*N* data available
Age at baseline, years (mean, s.d.)	59.1 (14.2)	66.8 (11.3)	56.7 (14.1)	267
Male sex, *n* (%)	63 (23.6)	19 (29.2)	44 (21.8)	267
Disease duration, years (median, IQR)	5.9 (1.9–15)	5.9 (2.4–13.1)	6.1 (1.9–16.2)	267
Smoking ever, *n* (%)	73 (27.3)	24 (36.9)	49 (24.3)	267
SSc autoantibodies, *n* (%)				
ACA	63 (23.8)	12 (18.5)	51 (25.5)	265
ATA	119 (44.7)	28 (43.1)	91 (45.3)	266
ARA	38 (14.8)	7 (11.7)	31 (15.7)	257
AntiPm/Scl	47 (18.2)	11 (18.3)	36 (18.2)	258
dcSSc, *n* (%)	92 (34.5)	27 (41.5)	65 (32.2)	267
Scleroderma pattern on NVC, *n* (%)	154 (74.8)	30 (73.2)	124 (75.2)	206
SRC, *n* (%)	6 (2.2)	4 (6.2)	2 (1)	267
Digital ulcers ever, *n* (%)	81 (30.3)	21 (32.3)	60 (29.7)	267
Pitting scars ever, *n* (%)	91 (34.1)	21 (32.3)	70 (34.7)	267
Puffy fingers, *n* (%)	186 (69.7)	36 (55.4)	150 (74.3)	267
Telangiectasia, *n* (%)	163 (61)	50 (76.9)	113 (55.9)	267
Joint involvement, *n* (%)	61 (22.8)	15 (23.1)	46 (22.8)	267
Increased inflammatory markers, *n* (%)	50 (18.7)	18 (27.7)	32 (15.8)	267
SCOpE score, (range 0–23) (mean, s.d.)	8.9 (4.6)	12.7 (4.4)	7.6 (3.9)	267
Dyspnoea NYHA ≥ II, *n* (%)	142 (53.2)	45 (69.2)	97 (48)	267
O_2_ saturation at rest (mean, s.d.)	97.3 (2.3)	96.1 (2.9)	97.6 (1.9)	233
PH on RHC, *n* (%)	26 (18.4)	15 (36.6)	11 (11)	141
FVC % (mean, s.d.)	86.6 (20.6)	84 (25.9)	91.1 (18.6)	256
DLCO/SB % (mean, s.d.)	65.7 (22.5)	51.5 (20.6)	69.8 (21.3)	258
6MWT, (mean, s.d.)	505 (121.5)	415.6 (124)	530.9 (108)	236
Immunosuppressants, ongoing at baseline, *n* (%)	48 (26.8)	14 (42.4)	34 (23.3)	179
Nintedanib ongoing at baseline, *n* (%)	5 (2.8)	0 0	5 (2.8)	179
Immunosuppressants, ever, *n* (%)	92 (51.4)	20 (60.6)	72 (49.3)	179
Nintedanib ever, *n* (%)	19 (10.6)	2 (6.1)	17 (11.6)	179

ATA: anti-topo I antibodies; ARA: anti-RNA polymerase III antibodies; antiPm/Scl: anti-Pm/scleroderma-antibodies; DLCO/SB%: diffusion of the lung for carbon oxide on single breath; FVC%: forced vital capacity; IQR: interquartile range; NVC: nailfold videocapillaroscopy; NYHA: New York Hearth Association; SCOpE: Scleroderma mOrtality p Eustar; SRC: scleroderma renal crisis; 6MWT: 6 min walking test.

**Table 2:** Distribution of parenchymal and vascular radiomics parameters among SSc patients with death or alive status at the end of follow-up (left) and with and without ILD progression (right).

	**Dead *n* (%) = 65 (24.3)**	**Alive *n* (%)** **=** **202 (75.7)**	**ILD progression *n* (%)** **=** **81 (50.6)**	**No ILD progression *n* (%)** **=** **79 (49.4)**
**Median (IQR)**	**TOTAL LUNGS**			
GGO	5.88 (0.29–26.06)	1.41 (0.27–5.04)	2.61 (0.54–7.39)	2.36 (0.52–9.72)
RET	2.23 (0.59–5.71)	0.75 (0.41–1.63)	0.81 (0.49–1.69)	1.01 (0.59–2.23)
HC	0.04 (0.03–0.15)	0.03 (0.02–0.07)	0.03 (0.01–0.07)	0.03 (0.02–0.1)
HL	0.93 (0.2–5.26)	2 (0.35–5.91)	1.78 (0.25–4.67)	2.14 (0.41–8.96)
Normal lung	76.43 (58.54–91.81)	92.37 (83.5–96.66)	91.70 (83.50–95.88)	88.54 (72.85–94.83)
PVV	2.9 (1.57–4.98)	1.65 (1.29–2.17)	1.78 (1.45–2.26)	1.81 (1.40–2.47)
**UPPER LUNG**				
GGO	0.74 (0.01–10.70)	0.03 (0–0.47)	0.08 (0–0.57)	0.13 (0–0.76)
RET	0.77 (0.03–4.51)	0.12 (0.02–0.42)	0.16 (0.02–0.37)	0.25 (0.05–0.70)
HC	0.01 (0–0.07)	0 (0–0.27)	0 (0–0.02)	0 (0–0.06)
HL	1.13 (0.24–2.64)	1.13 (0.39–5.88)	1.13 (0.22–7.47)	2.07 (0.49–5.85)
Normal lung	88.37 (75.68–98.1)	97.25 (90.11–98.85)	97.26 (90.44–98.85)	94.94 (74.26–97.94)
PVV	1.74 (1.04–3.37)	1.07 (0.92–1.45)	1.19 (1.01–1.35)	1.16 (0.95–1.58)
**MIDDLE LUNG**				
GGO	2.85 (0.49–25.26)	0.69 (0.25–2.84)	0.95 (0.43–3.21)	1.14 (0.43–4.95)
RET	1.93 (0.52–5.25)	0.70 (0.25–1.16)	0.70 (0.34–1.21)	0.70 (0.35–1.53)
HC	0.03 (0.02–0.10)	0.02 (0.01–0.04)	0.02 (0.01–0.05)	0.02 (0–0.04)
HL	0.87 (0.24–3.08)	1.37 (0.27–4.51)	1.37 (0.16–2.63)	1.52 (0.23–9.46)
Normal lung	86.08 (55.77–96.29)	94.99 (86.87–97.42)	95.46 (89.48–97.42)	90.08 (77.86–96.43)
PVV	3.11 (1.78–5.37)	2.11 (1.70–2.64)	2.18 (1.83–2.68)	2.24 (1.71–2.80)
**LOWER LUNG**				
GGO	11.31 (0.61–48.6)	3.55 (0.45–12.76)	6.16 (0.69–18.38)	6.11 (1.51–23.16)
RET	3.98 (1.21–11.10)	1.55 (0.79–3.45)	1.65 (1.02–3.67)	1.77 (0.93–4.47)
HC	0.07 (0.02–0.35)	0.05 (0.02-0.13)	0.07 (0.02–0.11)	0.05 (0.01–0.17)
HL	0.71 (0.12–4.60)	1.01 (0.11–4.59)	0.75 (0.08–4.58)	1.75 (0.12–4.6)
Normal lung	62.95 (22.02–93.07)	87.66 (72.63–93.96)	87.26 (77.67–93.96)	84.21 (56.67–93.37)
PVV	3.77 (1.40–7.06)	1.58 (1.14–2.28)	1.78 (1.27–2.46)	1.70 (1.24–2.83)

GGO: ground-glass opacities; HC: honeycombing; HL: hyperlucent; ILD: interstitial lung disease; PVV: pulmonary vessel volume; RET: reticular.

In multivariable Cox regression analysis including LTA radiomic features of the whole lung, the extents of PVV and honeycombing were associated with mortality. This result was confirmed in the sensitivity analysis looking at the three separate lung thirds (Supplementary Table S1). Conversely, when incorporating the extent of PVV in a multivariable model including ILD extent or fibrotic extent quantified by LTA, respectively, no independent radiomic predictor could be identified (data not shown).

**Figure 2:**
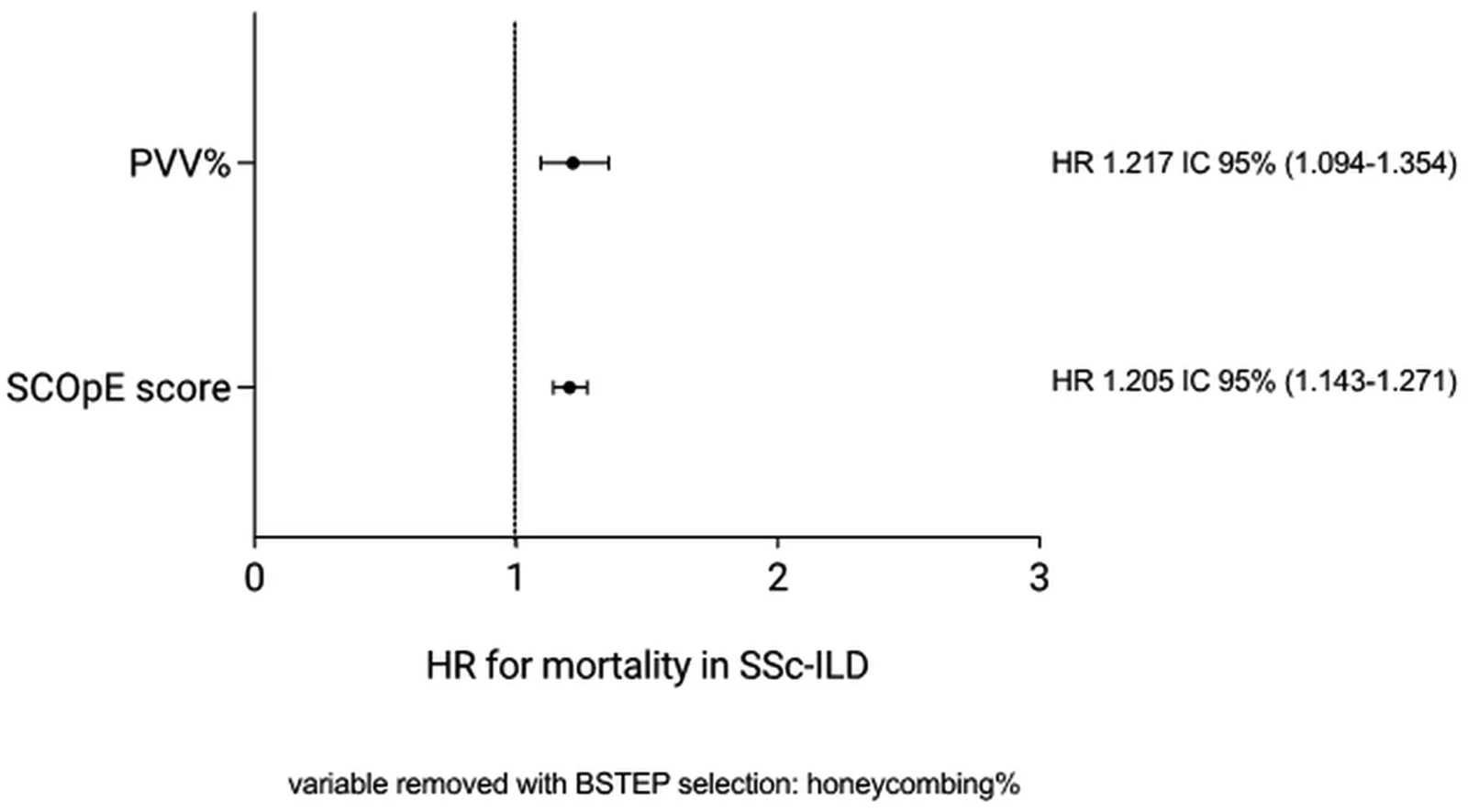
Forrest plot showing predictive radiomics extent of pulmonary vessel volume (PVV%) from whole lung and SCOpE score as predictors of SSc-ILD overall mortality. PVV: odds ratio; PVV: pulmonary vessel volume; SCOpE: Scleroderma mOrtality p Eustar; BSTEP: backword stepwise selection

When adjusting for the SCOpE score, PVV% was the only radiomic variable that remained significantly associated with mortality [hazard ratio (HR) 1.217 (1.094–1.354)] (Fig. 2). When exploring the anatomical distribution of the radiomic predictors, PVV% from the upper zones, but not from other zones, remained in the model to predict overall mortality, indicating a stronger association (Supplementary Fig. S1).

### ILD progression

Out of the study cohort, 160/267 (60%) patients had at least one annual follow-up available. Overall, a total of 261 annual visits over a median of 4.95 years of follow-up were included in the progression analyses. Of these patients, 81/160 (50.6%) experienced at least one episode of ILD progression (defined as decline in FVC of ≥5% or in DLCO of ≥10%), for a total count of 99/261 (37.9%) episodes of decline. This definition yielded the largest number of patients who progressed, and included all patients classified as progressors according to the other definitions tested.

Patients with progressive ILD had higher FVC and DLCO at baseline (Table 3), while LTA radiomic features were overall comparable with those of patients who did not progress, except for a slightly lower presence of reticulations among progressors (Table 2, right panel). In the multivariable GEE model testing LTA radiomic predictors from the whole lung, honeycombing, hyperlucent areas and reticular alterations were associated with ILD progression over 12 months. As for the mortality analysis, no significant radiomic predictors of progression were identified testing ILD extent or the fibrotic extent within the multivariable model.

**Table 3:** Clinical characteristics and functional features of SSc patients with and without ILD progression. Increased inflammatory markers are defined as CRP or ESR above the upper level of normality according to local laboratory. Immunosuppressants include CYC, MMF, rituximab, tocilizumab.

	Total *n* = 160	ILD progression *n* (%) = 81 (50.6)	No ILD progression *n* (%) = 79 (49.4)	*N* data available
Age at baseline, years (mean, s.d.)	58 (50–68)	57 (49.8–67.1)	59.8 (50–69)	159
Male sex, *n* (%)	35 (21.9)	18 (22.2)	17 (21.5)	160
Disease duration, years (median, IQR)	7.2 (2.7–15.8)	6.3 (2–17.6)	7.9 (3.5–15)	160
Smoking ever, *n* (%)	47 (29.4)	22 (27.2)	25 (31.6)	160
SSc autoantibodies, *n* (%)				
ACA	34 (21.4)	16 (19.8)	18 (23.1)	159
ATA	77 (48.1)	44 (54.3)	33 (41.8)	160
ARA	27 (17)	13 (16)	14 (17.9)	159
antiPm/Scl	30 (18.8)	17 (21)	13 (16.5)	160
dcSSc, *n* (%)	59 (36.9)	34 (42)	25 (31.6)	160
Scleroderma pattern on NVC, *n* (%)	98 (74.2)	50 (78.1)	48 (70.6)	132
SRC, *n* (%)	4 (2.5)	1 (1.2)	3 (3.8)	160
Digital ulcers ever, *n* (%)	62 (38.8)	25 (30.9)	37 (46.8)	160
Pitting scars ever, *n* (%)	66 (41.3)	30 (37)	36 (45.6)	160
Puffy fingers, *n* (%)	111 (69.4)	53 (65.4)	58 (73.4)	160
Telangiectasia, *n* (%)	97 (60.6)	51 (63)	46 (58.2)	160
Joint involvement, *n* (%)	41 (25.6)	21 (25.9)	20 (25.3)	160
Increased inflammatory markers, *n* (%)	31 (19.4)	14 (17.3)	17 (21.5)	160
Dyspnoea NYHA ≥ II, *n* (%)	82 (51.2)	44 (54.3)	38 (48.1)	160
O_2_ saturation at rest (mean, s.d.)	98 (96–99)	98 (96–99)	98 (96–99)	149
PH on RHC, *n* (%)	17 (21.5)	7 (15.2)	10 (30.3)	79
FVC % (mean, s.d.)	90 (74.3–101.8)	94 (79–102)	86 (69–98)	160
DLCO/SB % (mean, s.d.)	65.5 (50–79)	70 (58–83)	61 (47–74.5)	160
6MWT, (mean, s.d.)	540 (473.8–597.5)	543 (462–600)	540 (477–577)	150
Immunosuppressants, ongoing at baseline, *n* (%)	32 (26.9)	18 (32.7)	14 (21.9)	119
Nintedanib ongoing at baseline, *n* (%)	5 (4.2)	0 0	5 (7.8)	119
Immunosuppressants, ever, *n* (%)	83 (69.7)	37 (67.3)	46 (71.9)	119
Nintedanib ever, *n* (%)	19 (16)	10 (18.2)	9 (14.1)	119

ATA: anti-topo I antibodies; ARA: anti-RNA polymerase III antibodies; antiPm/Scl: anti-Pm/scleroderma-antibodies; DLCO/SB%: diffusion of the lung for carbon oxide on single breath; FVC%: forced vital capacity; ILD: interstitial lung disease; IQR: interquartile range; NVC: nailfold videocapillaroscopy; NYHA: New York Hearth Association; PH: pulmonary hypertension; RHC: right heart catheterization; SRC: scleroderma renal crisis; 6MWT: 6-minute walking test.

**Figure 3:**
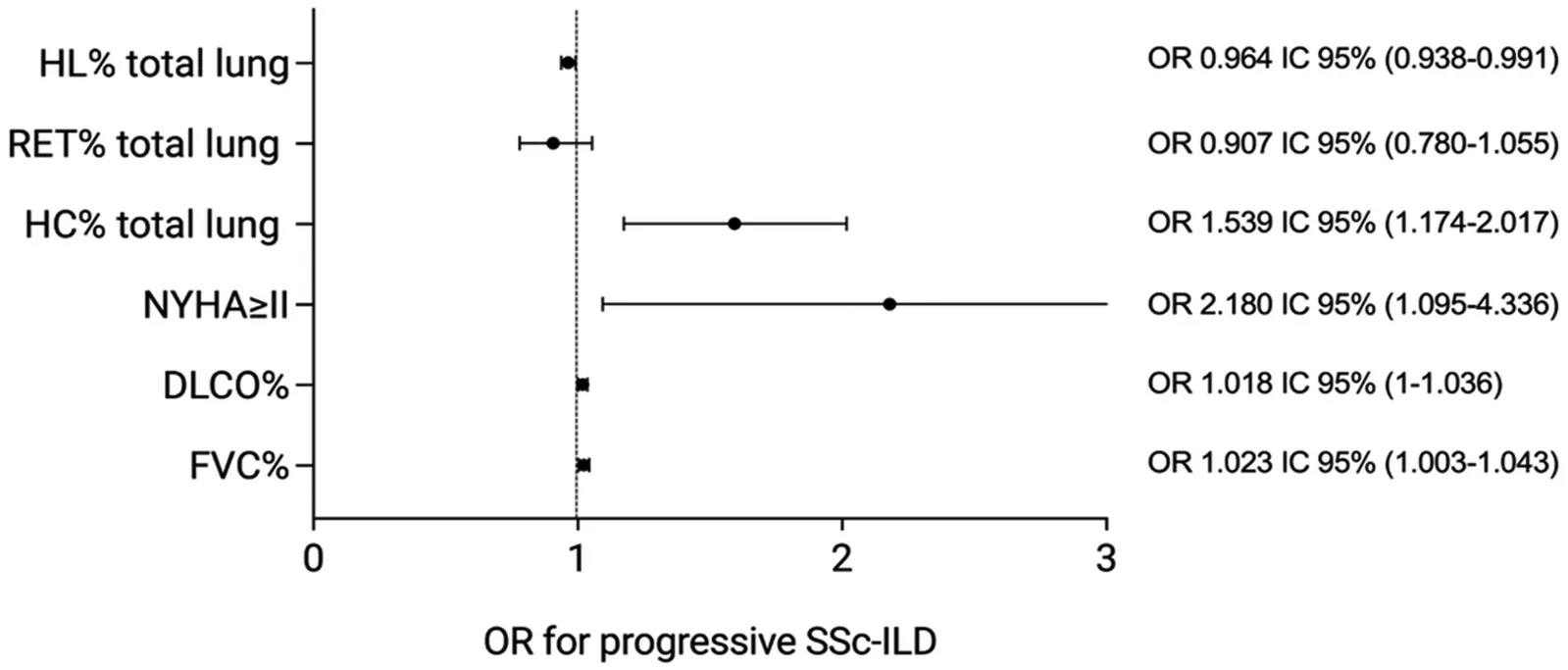
Forrest plot showing predictive radiomics features from whole lung and clinical risk factors for ILD progression. DLCO: diffusion of the lung for carbon oxide; FVC: forced vital capacity; HC: honeycombing; HL: hyperlucency; ILD: interstitial lung disease; NYHA: New York Hearth Association; PVV: odds ratio; RET: reticular

Among the clinical predictors, we identified DLCO%, FVC% and NYHA class ≥ II as independent risk factors (Supplementary Tables S2 and S3). When combining radiomics and clinical variables in the final GEE model, honeycombing extent [odds ratio (PVV) 1.539 (1.174–2.017)] was the only radiomic predictor retained, together with the three abovementioned clinical factors (Fig. 3).

As a secondary analysis, we explored the association of the anatomical distribution of the honeycombing across the three lung zones with the primary progression definition, but no significant association could be detected (Supplementary Fig. S2). When exploring the additional ILD progression criteria, none of the radiomic features was significantly associated with ILD progression, potentially due to the limited number of events recorded (Supplementary Table S4A–C).

## Discussion

In this study, evaluation of lung radiomics through texture-based analysis identified parameters associated with higher risk of both mortality and functional progression in SSc-ILD. Our data demonstrated the relevance of radiomics imaging analysis in the risk stratification of patients with SSc-ILD, further enriching the risk phenotype based on clinical and functional features.

Once ILD has been established, up to 67% of SSc patients progress at least once over 5 years [3], and this has a profound impact on survival [1, 27]. Despite relevant effort from the scientific community to date, the variability across studies has not yet allowed to identification and validation of comprehensive predictors of ILD progression, and the course of SSc-ILD remains largely unpredictable [7, 28]. Therefore, there is an urgent need to understand predictors driving poor outcomes, to enable timely therapeutic interventions.

Previous studies have used visual chest HRCT features as prognostic markers of SSc-ILD [9], with a major focus on ILD extent. So far, an extent of ILD on HRCT of ≥20% of lung tissue (or 10% to 20% combined with a FVC of <70%) has been considered the strongest parameter for predicting mortality [9, 29]. However, the difficulty in standardizing visual HRCT assessment, the required expertise, and the operator-dependent nature of the evaluation, contribute to potential heterogeneity in the application of the proposed measure. Additionally, many of these studies identified visual CT parameters through univariable analyses, thereby limiting the strength and generalizabilty of the results [9].

Our survival analysis identified the extent of PVV as the main predictor for all-cause mortality, independent of the contribution of clinical and functional risk factors. PVV% is a purely computer-based parameter that cannot be evaluated by the human eye and goes beyond the mere quantification of vascular components [30]. In fact, it reflects connected tubular structures of adjacent fibrotic regions, with the potential to detect pathological alterations within interstitial and vascular regions [31]. Previous studies in SSc have related higher extent of PVV values to the presence of ILD [13] and to its related functional impairment [14]. Additionally, previous findings from CTD-associated ILD, RA-ILD and IPF cohorts [15, 16, 30] are in line with our data, emphasizing the role of vascular alterations as a marker of severe ILD. It needs to be mentioned that, despite our consistent results, a direct relationship between PVV% and mortality specifically attributable to ILD cannot be established, as the available data did not include causes of death. Nevertheless, the observed increase in PVV and the fibrotic radiomic parameters in patients who subsequently died support our findings and the face validity of PVV% as a marker of ILD severity and mortality risk. Previous studies have found that PVV% is more prominent in areas of spared, unaffected lung tissue, possibly related to the increased pressure on vascular structures in more fibrotic regions [14, 31], leading to a perfusion redistribution. Since lung bases are usually the most involved areas in SSc-ILD [32], this supports our exploratory results, showing upper lung PVV% as the specific feature associated with increased mortality risk.

Our findings from the progression analysis contrast with previous data in which the extent of GGO% predicted functional decline [33]. Ferrazza *et al.* directed their analysis towards the delta % changes of lung function parameters within 12 months, while we adopted categorical definitions of functional progression based on international guidelines and expert consensus, and also included multiple assessments over a longer follow-up period.

Notably, although the extent of honeycombing did not significantly differ at baseline HRCT between progressors and non-progressors, it was still associated with ILD progression, reflecting our longitudinal modelling approach and potentially capturing the dynamic evolution of fibrosis. In our study, the first HRCT included in the model was the first suitable for radiomic analysis, not necessarily the first performed at ILD onset. This approach further supports the relevance of our observations, as it may have included patients who had already progressed before inclusion and then stabilized [27], which could explain the lower baseline FVC and DLCO values observed in non-progressors. In contrast, modelling alterations within the ILD extent and fibrotic extent variables did not yield the same results, likely due to a dilution effect.

Some previous studies on visual CT have proposed the usual interstitial pneumonia (UIP) pattern as a predictor of SSc-ILD progression [34, 35], but this has not been consistently confirmed in the literature. Our results may be in line with this hypothesis, identifying honeycombing—the hallmark of the UIP pattern—as a key factor associated with ILD progression. Although radiomics allows an objective, highly sensitive, and reproducible quantification of lung texture, detecting changes potentially even when not appreciable by visual assessment, it does not report certain information provided by the visual evaluation. Whether the radiological ILD phenotype at visual assessment may have influenced our results remains therefore to be determined [36, 37].

Our study should be interpreted within its limitations. First, the overall number of patients remains limited. In addition, the definitions of ILD progression we applied relied solely on functional criteria, not incorporating radiological worsening, which is also part of the current criteria for defining progressive ILD [36]. Moreover, we did not adjust our analysis for the use of immunosuppressive, vasoactive, or nintedanib therapy, given the limited number of events we recorded in our study population and the nature of our long-term observational data collection, spanning over 20 years, therefore reflecting changing practice. Additionally, we could not exclude a certain length-time bias in the mortality analysis, as more severe patients tended to display a worse outcome. Overall, radiomic-based analyses are not yet widely implemented in routine clinical practice worldwide, and some techniques previously applied in SSc-ILD, such as CALIPER, are not freely available, which may limit the generalizability and clinical applicability of such approaches. Finally, while GEE models were selected to evaluate population-averaged longitudinal associations while accounting for repeated measures within patients, mixed-effects models could provide additional information on subject-specific progression trajectories. Future studies with more standardized follow-up schedules may benefit from the application of mixed-effects approaches to further characterize individual disease evolution.

However, our study relies on relevant strengths, consisting of the detailed clinical and functional available data, the longitudinal design, and the availability of multiple follow-up assessments. Radiomic analysis performed during both earlier and later phases of ILD, even for the same patients, supports the generalizability of our results to different ILD scenarios. Finally, we were able to determine the contribution of the radiomics features towards the prediction of ILD prognosis, adjusted and corrected for the already known clinical risk factors, supporting their independent role and additional value in clinical care and research.

## Conclusion

Our findings underscore the role of radiomics in the identification of patients at higher risk of ILD progression and mortality, supporting its potential for risk stratification in clinical practice and for enriching target populations in clinical trials.

All participants gave written informed consent. The study was conducted upon ethical approval (Cantonal Ethics Committee Zurich, BASEC Nr.2016–01515 and BASEC-Nr.2018–02165) and in accordance with the Declaration of Helsinki.

## Supplementary Material

keag463_Supplementary_Data

## Data Availability

Data are available from the corresponding author upon request.
