# Supplementary material for: Radiomic analysis of high-resolution computed tomography predicts interstitial lung disease progression and mortality in systemic sclerosis
Source: Rheumatology (Oxford). 2026 Aug 27;65(9):keag463. doi: 10.1093/rheumatology/keag463 (PMC13592468; doi:10.1093/rheumatology/keag463)
Supplement: Supplementary file 1 [file keag463_supplementary_data.docx]

**Supplementary material**

**Supplementary Table S1.** Association of the radiomic features from the whole lung and upper, middle and lower zones with mortality

| **%** | **LTA TOT**  **HR (CI 95%)** | **UPPER**  **HR (CI 95%)** | **MIDDLE**  **HR (CI 95%)** | **LOWER**  **HR (CI 95%)** |
| --- | --- | --- | --- | --- |
| **PVV** | 1.389 (1.268-1.522) | 1.437 (1.280-1.613) | 1.178 (1.082-1.282) | 1.191(1.098-1.291) |
| **GGO** | 1.003 (0.985-1.021) | 1.001 (0.983-1.020) | 1.006 (0.988-1.025) | 1.004 (0.988-1.020) |
| **RET** | 1.000 (0.952-1.049) | 1.024 (0.997-1.051) | 1.035 (1.008-1.063) | 1.019 (1.006-1.031) |
| **HL** | 1.017 (0.998-1.035) | 1.010 (0.994-1.027) | 1.008 (0.989-1.027) | 1.023 (1.004-1.042) |
| **HC** | 1.262 (1.010-1.576) | 1.460 (1.070-1.990) | 1.313 (1.088-1.584) | 1.022 (0.876-1.192) |

CI: confidence interval; GGO: ground glass opacities; HC: honeycombing; HL: hyperlucent; HR: hazard ratio; PVV: pulmonary vessel volume; RET: reticular

**Supplementary Table S2.** Association of the radiomic features from the whole lung and upper, middle and lower zones with progression according to the functional domain criteria of the PPF-ILD definition

| **%** | **LTA TOT**  **OR (CI 95%)** | **UPPER**  **OR (CI 95%)** | **MIDDLE**  **OR (CI 95%)** | **LOWER**  **OR (CI 95%)** |
| --- | --- | --- | --- | --- |
| **PVV** | 0.896 (0.622-1.289) | 0.946 (0.638-1.402) | 0.859 (0.604-1.222) | 0.925 (0.755-1.134) |
| **GGO** | 1.020 (0.977-1.064) | 1.002 (0.961-1.045) | 1.017 (0.973-1.064) | 1.012 (0.992-1.034) |
| **RET** | 0.828 (0.689-0.996) | 0.953 (0.816-1.113) | 892 (0.698-1.140) | 0.909 (0.821-1.007) |
| **HL** | 0.970 (0.948- 0.992) | 0.974 (0.958-0.991) | 0.965 (0.942-0.990) | 0.943 (0.911-0.975) |
| **HC** | 1.602 (1.176-2.183) | 1.377 (0.997-1.902) | 2.360 (1.079-5.160) | 1.145 (1.038-1.263) |

CI: confidence interval; GGO: ground glass opacities; HC: honeycombing; HL: hyperlucent; OR: odds ratio; PVV: pulmonary vessel volume; RET: reticular

**Supplementary Table S3.** Association of the clinical features with progression according to the functional domain criteria of the PPF-ILD definition

|  | **OR (CI 95%)** |
| --- | --- |
| **Age** | 1.003 (0.981-1.026) |
| **Sex** | 1.343 (0.690-2.616) |
| **DLCO/SB %** | 1.019 (1.002-1.036) |
| **FVC %** | 1.023 (1.005-1.041) |
| **Disease duration** | 0.977 (0.971-1.024) |
| **NYHA class ≥ II** | 2.038 (1.073-3.870) |
| **dcSSc** | 1.637 (0.934-2.869) |

CI: confidence interval; dcSSc: diffuse cutaneous systemic sclerosis; DLCO/SB%: diffusion of the lung for carbon oxide on single breath; FVC%: forced vital capacity; NYHA: New York Hearth Association; OR: odds ratio

**Supplementary Table S4.** Generalized estimated equations (odds ratio and 95% confidence interval) showing the lack of predictive ability of the radiomic features - both for the whole lung and across the upper, middle, and lower lung zones - when applying different definitions of ILD progression.

| **%** | **LTA TOT**  **OR (CI 95%)** | **UPPER**  **OR (CI 95%)** | **MIDDLE**  **OR (CI 95%)** | **LOWER**  **OR (CI 95%)** |
| --- | --- | --- | --- | --- |
| **PVV** | 1.073 (0.726-1.586) | 1.198 (0.716-1.684) | 1.027 (0.692-1.525) | 0.940 (0.773-1.142) |
| **GGO** | 1.005 (0.960-1.053) | 0.997 (0.951-1.045) | 1.004 (0.952-1.058) | 1.011 (0.989-1.033) |
| **RET** | 0.874 (0.730-1.046) | 0.965 (0.786-1.184) | 0.914 (0.752-1.112) | 0.932 (0.838-1.036) |
| **HL** | 0.984 (0.962-1.006) | 0.991 (0.974-1.008) | 0.979 (0.955-1.005) | 0.948 (0.911-0.987) |
| **HC** | 1.215 (0.721-2.049) | 0.524 (0.060-4.616) | 1.512 (0.555-4.119) | 1.146 (1.019-1.289) |

**IV-A) FVC absolute decline ≥ 5%.** Analysis included 55/160 (34.4%) patients over 62/261 (23.8%) episodes of progression

| **%** | **LTA TOT**  **OR (CI 95%)** | **UPPER**  **OR (CI 95%)** | **MIDDLE**  **OR (CI 95%)** | **LOWER**  **OR (CI 95%)** |
| --- | --- | --- | --- | --- |
| **PVV** | 1.068 (0.767-1.489) | 1.108 (0.685-1.794) | 1.003 (0.717-1.403) | 0.929 (0.764-1.131) |
| **GGO** | 1.002 (0.961-1.044) | 0.999 (0.946-1.055) | 1.004 (0.964-1.047) | 1.012 (0.990-1.034) |
| **RET** | 0.972 (0.805-1.173) | 1.007 (0.806-1.258) | 1.019 (0.835-1.243) | 0.983 (0.909-1.063) |
| **HL** | 0.964 (0.916-1.013) | 0.987 (0.962-1.013) | 0.968 (0.920-1.018) | 0.902 (0.812-1.000) |
| **HC** | 1.121 (0.806-1.557) | 0.477 (0.035-6.490) | 1.460 (0.731-2.918) | 1.109 (1.019-1.207) |

**IV-B)** **FVC relative decline ≥ 10 % or FVC 5-9% and DLCO relative decline ≥ 15%.** Analysis included 34/160 (21.3%) patients over 38/261 (14.5%) episodes of progression

| **%** | **LTA TOT**  **OR (CI 95%)** | **UPPER**  **OR (CI 95%)** | **MIDDLE**  **OR (CI 95%)** | **LOWER**  **OR (CI 95%)** |
| --- | --- | --- | --- | --- |
| **PVV** | 1.196 (0.776-1.843) | 1.315 (0.638-2.711) | 1.145 (0.714-1.838) | 0.963 (0.795-1.166) |
| **GGO** | 0.970 (0.926-1.017) | 0.959 (0.886-1.038) | 0.974 (0.923-1.029) | 1.003 (0.981-1.025) |
| **RET** | 1.094 (0.946-1.265) | 1.099 (0.918-1.317) | 1.130 (0.944-1.354) | 1.011 (0.952-1.073) |
| **HL** | 0.975 (0.935-1.016) | 0.993 (0.968-1.018) | 0.978 (0.936-1.022) | 0.921 (0.842-1.007) |
| **HC** | 1.064 (0.790-1.434) | 0.469 (0.031-7.161) | 1.250 (0.664-2.350) | 1.107 (1.028-1.191) |

**IV-C)** **FVC relative decline ≥ 10 %.** Analysis included 29/160 (18.1%) patients over 30/261 (11.5%) episodes of progression

CI: confidence interval; GGO: ground glass opacities; HC: honeycombing; HL: hyperlucent; OR: odds ratio; PVV: pulmonary vessel volume; RET: reticular

**Supplementary Figure S1.** Forrest plot showing the predictive value of extent of pulmonary vessel volume (PVV%) according the anatomical distribution for the overall morality

OR: Odds Ratio; PVV: pulmonary vessel volume; SCOpE: Scleroderma mOrtality p Eustar

**Supplementary Figure S2.** Forrest plot showing the lack of predictive value of HC% according to the anatomical distribution for ILD progression

DLCO: diffusion of the lung for carbon oxide; FVC: forced vital capacity; HC: honeycombing; NYHA: New York Hearth Association; OR: Odds Ratio
